# Reference Intervals of Total Testosterone in Adult Filipino Men

**DOI:** 10.1155/2020/8877261

**Published:** 2020-11-28

**Authors:** Myrna Buenaluz‐Sedurante, Mark Isaiah K. Co, Daryl Jade T. Dagang, Racquel G. Bruno, Annie Jane N. Sarmiento, Michael L. Tee

**Affiliations:** ^1^Department of Physiology, University of the Philippines College of Medicine, Manila, Philippines; ^2^Division of Endocrinology, Diabetes and Metabolism, Department of Medicine, University of the Philippines College of Medicine, Manila, Philippines

## Abstract

**Background:**

The reference range of total testosterone needs to be established locally as ethnic differences in adiposity, insulin sensitivity, and sex hormone-binding globulin (SHBG) levels may affect total testosterone levels. The aim of this study is to establish the reference intervals of total testosterone from healthy, young adult Filipino males.

**Methods:**

The study included 110 healthy, Filipino male volunteers aged 21–40, studying or working at the University of the Philippines Manila. Clinical history, height, weight, body mass index (BMI), and blood pressure (BP) were obtained, and blood for total testosterone, SHBG, albumin, insulin, fasting blood sugar (FBS), and total cholesterol was collected. Free testosterone was calculated using Vermeulen's formula. The 2.5th to 97.5th percentiles of subjects for total testosterone were used as the normative range for Filipino men.

**Results:**

The reference range of total testosterone is 7.33–53.01 nmol/L.

**Conclusion:**

The present study derived reference ranges of total testosterone using data from apparently healthy, young adult men to support clinical services.

## 1. Introduction

Androgens are vital for the male's physical attributes, distinct strength, behavior, and reproduction [1]. Male androgens mostly come from the testes in the form of testosterone and dihydrotestosterone. The adrenals also contribute a small portion in the form of dehydroepiandrosterone (DHEA) and dehydroepiandrosterone sulfate (DHEA-S). These adrenal hormones can also be converted into testosterone in the periphery. Testosterone levels may decline with age [2, 3]. In addition, androgen deficiency has been associated with many diseases such as obesity, type 2 diabetes mellitus, metabolic syndrome, depression, and Alzheimer's disease, human immunodeficiency virus (HIV) [4–8] and with the use of certain medications such as steroids, anticonvulsants, and opioids [1]. Androgen deficiency causes osteoporosis, increased cardiovascular mortality, and poor quality of life [1, 9, 10].

To determine whether a patient is testosterone deficient, guidelines of the European Association of Urology (EAU) and the Endocrine Society (ES) recommend that a clinician considers clinical signs and symptoms in conjunction with two early morning total testosterone values as levels peak in the morning [11, 12]. If both values are consistently low, then this establishes the diagnosis. If the clinical presentation is associated with a low normal total testosterone, then free testosterone levels should be analyzed via equilibrium dialysis or calculated with the help of sex hormone-binding globulin (SHBG). Recall that, in young adult men, total testosterone is composed of the following fractions: 2% in the free form, 68% weakly bound to albumin, and 30% tightly bound to SHBG [13]. When tightly bound to SHBG, testosterone is not biologically available. Concentrations of SHBG vary widely in healthy men and are related to variables such as diet, body mass index (BMI), insulin concentration, smoking, and age [14]. Thus, ideally, free, and nonspecifically bound testosterone would generally reflect the clinical condition more accurately than total testosterone. However, tests for free (by equilibrium dialysis) and bioavailable testosterone (via ammonium precipitation) are nonautomated, are time-consuming, require expensive techniques, and therefore are not routinely performed in most laboratories. Direct radioimmunoassay, while available, has been criticized for the lack of accuracy [15, 16]. Alternatively, the concentrations of free testosterone and bioavailable testosterone can be calculated by the use of one of several published algorithms. These algorithms assume that when the concentrations of total testosterone, SHBG, and albumin and the constants for the binding of testosterone to SHBG and albumin are known, free testosterone and bioavailable testosterone can be calculated [17, 18].

Racial differences in circulating total testosterone levels have been noted due to varying SHBG levels resulting from ethnic differences in adiposity and insulin sensitivity. Thus, normative values for Whites have been determined in many countries such as Germany, the United States, Wales, and Britain [19–23] using various sampling techniques and laboratory methods. So far, no reference values for African Americans have been generated as most studies comparing African Americans and Whites showed that their testosterone levels are not significantly different [24, 25]. Reference values for East Asians have also been published [26, 27]. There are no reference ranges published for Southeast Asians, that is, those who reside in the Philippines, Singapore, Malaysia, Indonesia, and Brunei. This study aims to provide reference data for the definition of normal total testosterone levels in young, healthy, nonobese, Filipino males. In addition, since adiposity decreases insulin sensitivity and this in turn decreases SHBG, total testosterone will be correlated with waist-hip ratio (WHR), SHBG, and insulin levels. The data may then be used to diagnose androgen deficiency among this group.

## 2. Methods

### 2.1. Study Design

This cross-sectional study was conducted according to a protocol approved by the Technical Review Board of the University of the Philippines, Department of Physiology, and the University of the Philippines Manila Ethics Review Board. This study used the Strengthening the Reporting of Observational Studies in Epidemiology (STROBE) cross-sectional reporting guidelines [28].

### 2.2. Reference Sample Population

This study invited all healthy, young adult males aged 21–40 years, studying or working at the University of the Philippines Campus in Manila and its affiliated hospital, Philippine General Hospital, from 2016 to 2019 through personal letters of invitation and advertisements. This sampling assumed that healthy members of the said local community adequately represent the population of the country. The sample size was based on studies conducted by Lott and Mitchell [29] and echoed recently by Wellek, et al. [30], which state that a sample size of 110 for normally distributed data and 119 for non-Gaussian distributions will result in stable 2.5% and 97.5% reference limits at the 95% confidence level.

The study followed the International Federation of Clinical Chemistry's (IFCC) Expert Panel Recommendations on the Theory of Reference Values which advise a priori selection of participants by exclusion of specific physiologic, pathologic, and lifestyle conditions which contribute to biological variability [31, 32]. Thus, subjects with the following criteria were excluded from the study: obesity defined as a BMI ≥25; fasting blood sugar (FBS) ≥126 mg/dl; hypertension (BP ≥140/90 mmHg); hypercholesterolemia (total cholesterol ≥240); self-reported history of diabetes, osteoporosis, chronic lung disease, ulcer, HIV, cancer, cerebrovascular disease, myocardial infarction, stroke, congestive heart failure, bypass, angioplasty, claudication, hyperthyroid or hypothyroid disease, and infertility; current use of prescription medication, including eye drops, topical medications, and inhalers; history and present intake of testosterone, steroids, opioids, anticonvulsants, and male fertility agents; smoking (present or past); alcohol consumption exceeding 600 ml ethanol per week, corresponding to approximately six drinks per day, one drink being 15 ml ethanol (10 oz. beer, 4 oz. wine, or 1.5 oz. spirits); subject works in shifts; and lastly, a family history of hypogonadism or infertility (self-reported).

### 2.3. Data Collection Procedure

Informed consent was obtained after which subjects were interviewed regarding birthdate, past illnesses, current medications, smoking and alcoholic beverage intake, and family illnesses, and then the following were obtained: height, weight, waist circumference, hip circumference, and blood pressure. Height was measured without shoes using a portable stadiometer and recorded to the nearest 0.1 cm. Weight in light clothing without shoes was recorded to the closest 0.1 kg. Waist circumference was measured using a soft measuring tape midway between the lowest rib margin and the iliac crest in a standing position. Hip circumference was measured at the widest part of the gluteal region. Blood pressure was measured using a calibrated aneroid sphygmomanometer. Blood sample was collected in red-top tubes after a 10 hour fast, within 4 hours of awakening.

### 2.4. Assays

FBS, albumin, and total cholesterol were analyzed using a Cobas Integra 400Plus clinical chemistry analyzer. Total testosterone levels were measured with the Testosterone [I-125] RIA Kit (RK-61CT-Institute of Isotopes Ltd., Budapest). The inter- and intra-assay coefficient of variations (CV) were 12% and 8.9%, respectively. SHBG levels were determined using the SHBG [I-125] IRMA Kit (RK-86CT-Institute of Isotopes Ltd., Budapest). The inter- and intra-assay coefficients of variation (CV) were 6.04% and 8.58%. Lastly, insulin levels were measured using the Insulin [1-125] IRMA Kit (RK-400CT-Institute of Isotopes Ltd., Budapest). The inter- and intra-assay coefficients of variation (CV) were less than 17.1% and 4.4%, respectively. For the testosterone assay, a zero calibrator, 5 calibrators, and 2 quality control test samples were used, while for the insulin and SHBG assay, the same number of calibrators with 1 quality control test sample was used.

Free testosterone was calculated using the algorithm developed by Vermeulen [17]. This algorithm is available as an online calculator provided by the International Society for the Study of the Aging Male (ISSAM) [33].

### 2.5. Data Analysis

Data were entered in Excel, and descriptive statistics were then calculated. Missing data were managed by case deletion. RefVal 4.11 (Solberg, Oslo, Norway), a program developed by the IFCC to calculate reference intervals, was used [34]. The program defines the reference interval as the interval of values containing the central 95% of a healthy population. As part of the program, nonnormally distributed data are transformed. The two-stage transformation utilizes the Manly exponential transformation to remove skewness as its first stage and the modulus function of John and Draper as the final stage to remove the remaining kurtosis of the distribution. If the normality assumptions are still not met after the transformation, a nonparametric bootstrap method is employed where reference intervals are estimated over large numerous resamples. For this study, the program calculated reference intervals parametrically. Data are expressed as mean ± SD, median, and range at 2.5th to 97.5th percentiles.

In addition to using RefVal, nonparametric correlation tests using Spearman's rank rho coefficient were used to assess the association between variables.

## 3. Results

A total of 190 males volunteered to participate in the study, but 71 were excluded due to the history of smoking and increased BMI. One hundred and nineteen were eventually enrolled. Five participants were excluded due to incomplete data, while 4 were excluded due to increased FBS or cholesterol. The final study sample included 110 males with age between 21 and 40. The mean age and SD were 27.49 and 5.33 years. Anthropometric measures showed a mean and SD of BMI of 22.32 and 1.96 kg/m^2^ and a waist-hip ratio (WHR) of 0.92 and 0.05.  Table 1 presents the demographic profile and anthropometric profile of these healthy, young adult Filipino males. The reference range for total testosterone is 7.33–53.01 nmol/L, with a mean, SD of testosterone of 22.68 nmol/L, 12.37 and a median of 18.61 nmol/L. Calculated free testosterone showed a mean, SD of 0.55 nmol/L, 0.33 with a median of 0.44 nmol/L. Regarding distribution, total testosterone levels were skewed to the right. The serum insulin levels were also skewed to the right with an evident outlier that was also detected by Dixon's algorithm. The distribution of sex hormone-binding globulin concentration was almost symmetrical. After transformation, total testosterone, insulin, and SHBG levels assumed a Gaussian distribution. See Supplementary Figures S1–S6in Supplementary Materials for full image analysis.

The correlation between total testosterone, insulin, WHR, and SHBG is shown in Table 2. Insulin was positively correlated with WHR and negatively correlated with SHBG. Total testosterone was positively correlated with SHBG.

## 4. Discussion

We developed reference ranges for young, nonobese Filipino males aged 21–40. There are no reported reference intervals for other predominantly Malay groups such as those from Indonesia, Thailand, Brunei, nor Singapore, although there is an article comparing average testosterone levels between Malays and Chinese men in Malaysia using chemiluminescence enzyme immunoassay (CLEIA) [35]. This study written by Chin and colleagues reported a mean of 20.4 nmol/L with a standard deviation of 6.4 nmol/L. This is comparable with our results, with a mean of 21.86 nmol/L and a standard deviation of 10.8 nmol/L.  Table 3 is a comparison of reference ranges for various ethnic groups. The lower limit of our study's reference range is closest to that established by Iwamoto et al. for the Japanese cohort [27]. Our results also span the range of normative values for most ethnic groups.

Increased adiposity causes insulin resistance resulting in a subsequent rise in insulin levels. Insulin resistance decreases SHBG levels resulting in lower total testosterone levels [14]. This is echoed by our study where insulin levels were positively correlated with WHR and negatively correlated with SHBG. However, insulin was not negatively correlated with total testosterone as might have been expected. To further investigate this last analysis, we then calculated Homeostatic Model Assessment of Insulin Resistance (HOMA IR), a standard measure of insulin resistance, and correlated it with total testosterone. This post hoc analysis did not reveal a significant correlation either, probably due to small sample size. Lastly, the study also showed that total testosterone and SHBG were positively correlated.

Our study has several strengths. First, stringent criteria were used to select subjects, excluding the obese, smokers, and diseases or conditions which may affect total testosterone levels. Second, a single technician using meticulous quality control procedures performed the assays.

The study has several limitations. First, our study sample is not population-based but instead tested healthy volunteers. However, the ICC deems samples from both groups as valuable and acceptable. If we look at the population-based compared to healthy volunteer studies in Whites, as tabulated in Table 3, note that the 2 population-based studies, the first by Freidrich and the second by Travison, both showed comparable results with three of the four nonpopulation-based studies [21–24]. Incidentally, note also that the results were similar despite differing laboratory techniques (CLEIA and LS-MS). Second, we generated reference intervals for young adult men only following the approach of Bhasin and colleagues [36]. Reference intervals are generated from this age group for parameters that exhibit substantial age-related changes such as *T*-scores in bone mineral density and reference values for muscle strength and physical performance in sarcopenia.

In conclusion, we generated reference ranges for total testosterone specific for Filipinos using data from apparently healthy men.

## Figures and Tables

**Table 1:** Characteristics of study participants.

	Mean	Standard deviation
Age (years)	27.49	5.33
Height (m)	168.59	6.37
Weight (kg)	63.74	7.62
BMI^a^ (kg/m^2^)	22.32	1.96
Waist-hip ratio	0.92	0.05
SBP^b^ (mmHg)	115.38	10.93
DBP^c^ (mmHg)	73.21	8.63
FBS^d^ (mg/dl)	86.29	7.46
Total cholesterol (mg/dl)	179.91	35.53
Albumin (g/l)	49.21	2.44
Insulin (mIU/ml)	11.08	8.9
HOMA2 IR^e^	1.36	0.85
SHBG^f^ (nmol/L)	24.46	10.6

^a^Body mass index; ^b^systolic blood pressure; ^c^diastolic blood pressure; ^d^fasting blood sugar; ^e^homeostatic model assessment of insulin resistance; ^f^sex hormone-binding globulin.

**Table 2:** Spearman's rho correlation between testosterone, SHBG, and insulin levels.

*X*	*Y*	Spearman's rho	*p* value	Correlation
Insulin	WHR	0.27	0.004	Weak positive
Insulin	SHBG	−0.36	0.001	Moderate negative
Insulin	Total testosterone	0.08	0.392	Insignificant
Total testosterone	HOMA2 IR^a^	0.08	0.419	Insignificant
Total testosterone	SHBG^b^	0.21	0.024	Weak positive

^a^Homeostatic model assessment of insulin resistance; ^b^sex hormone-binding globulin.

**Table 3:** Reference ranges for total testosterone in young adult males across different ethnic groups.

Study author	Race	Assay	*N*	95% range (nmol/l)
Population-based				
Friedrich	White	CLEIA^a^	416	10.4–32.3
2008 (20)	(Germany)			
Travison	White	LC-MS^b^	400	9.16–31.78
2017 (24)	(US and Europe)			
Shen	East Asian	CLEIA	227	13.61–19.28
2013 (26)	(China)			
Iwamoto	East Asian	RIA	1172	6.96–26.00
2004 (27)	(Japan)			
Healthy volunteers				
Shatzl	White	CLEIA	133	10.4–28.8
2003 (21)	(Austria)			
Boyce	White	RIA^c^	199	10.04–38.76
2004 (22)	(UK)			
Elmlinger	White	CLEIA	446	8.7–29.6
2005, (23)	(Germany)			
Neale	White	LC-MS	67	10.6–31.9
2013 (24)	(Wales)			

^a^Chemiluminescence enzyme immunoassay; ^b^liquid chromatography-mass spectrometry; ^c^radioimmunoassay.

## Data Availability

The data that support the findings of this study are available from the corresponding author upon reasonable request.

## Supplementary Materials

Supplementary Materials Figure 1: distribution of nontransformed data for total testosterone. Figure 2: distribution of nontransformed data for serum insulin. Figure 3: distribution of nontransformed data for sex hormone-binding globulin. Figure 4: distribution of transformed data for total testosterone. Figure 5: distribution of transformed data for serum insulin. Figure 6: distribution of transformed data for sex hormone-binding globulin. http://downloads.hindawi.com/journals/ije/2020/8877261.f1.zip.
