# Supplementary material for: Reference Intervals of Total Testosterone in Adult Filipino Men
Source: Int J Endocrinol. 2020 Nov 28;2020:8877261. doi: 10.1155/2020/8877261 (PMC7719543; doi:10.1155/2020/8877261)
Supplement: Supplementary Materials — Figure 1: distribution of nontransformed data for total testosterone. Figure 2: distribution of nontransformed data for serum insulin. Figure 3: distribution of nontransformed data for sex hormone-binding globulin. Figure 4: distribution of transformed data for total testosterone. Figure 5: distribution of transformed data for serum insulin. Figure 6: distribution of transformed data for sex hormone-binding globulin. http://downloads.hindawi.com/journals/ije/2020/8877261.f1.zip. [file 8877261.f1.docx]

SUPPLEMENTARY FIGURES FOR

Reference Intervals of Total Testosterone in Adult Filipino Men

Myrna Buenaluz Sedurante^1^, Mark Isaiah K. Co^2,^ Daryl Jade T. Dagang^2,^ Racquel G. Bruno^2,^ Annie Jane N. Sarmiento^2,^ Michael L. Tee^1^

^1^Department of Physiology, University of the Philippines College of Medicine, Manila; ^2^Division of Endocrinology, Diabetes and Metabolism, Department of Medicine, University of the Philippines College of Medicine, Manila, Philippines


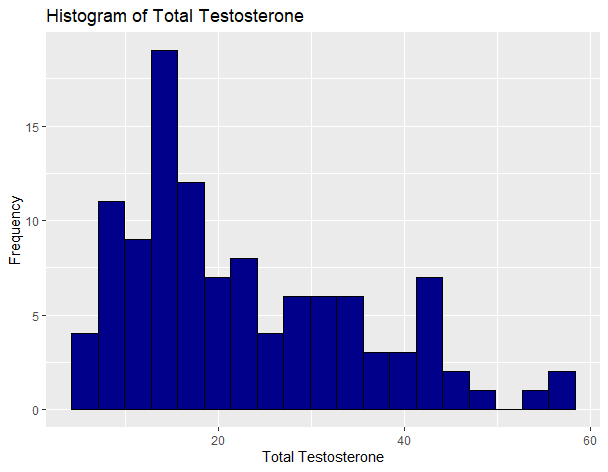


Supplementary Figure 1: Distribution of Non-Transformed Data for Total Testosterone


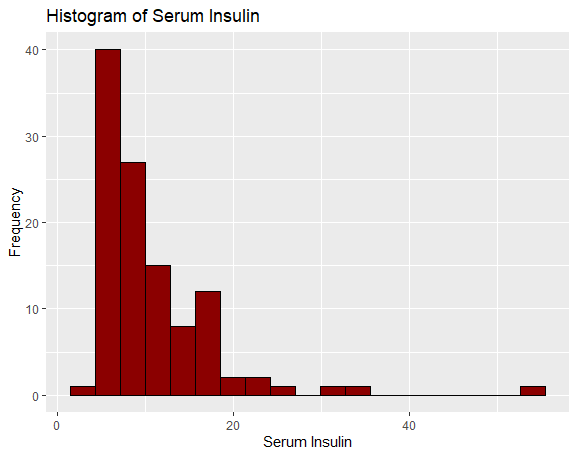


Supplementary Figure 2: Distribution of Non-Transformed Data for Insulin


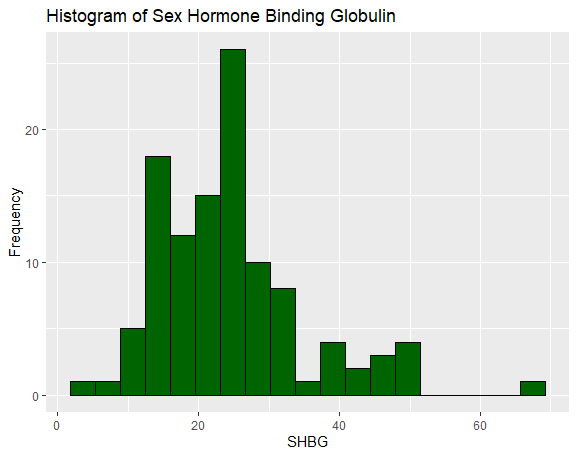


Supplementary Figure 3: Distribution of Non-Transformed Data for Sex Hormone Binding Globulin


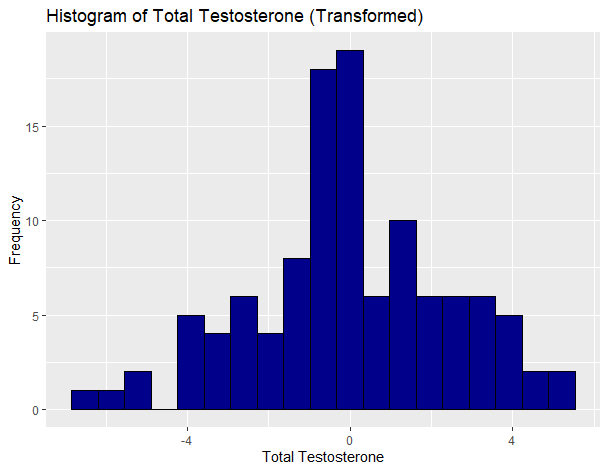


Supplementary Figure 4: Distribution of Transformed Data for Total Testosterone


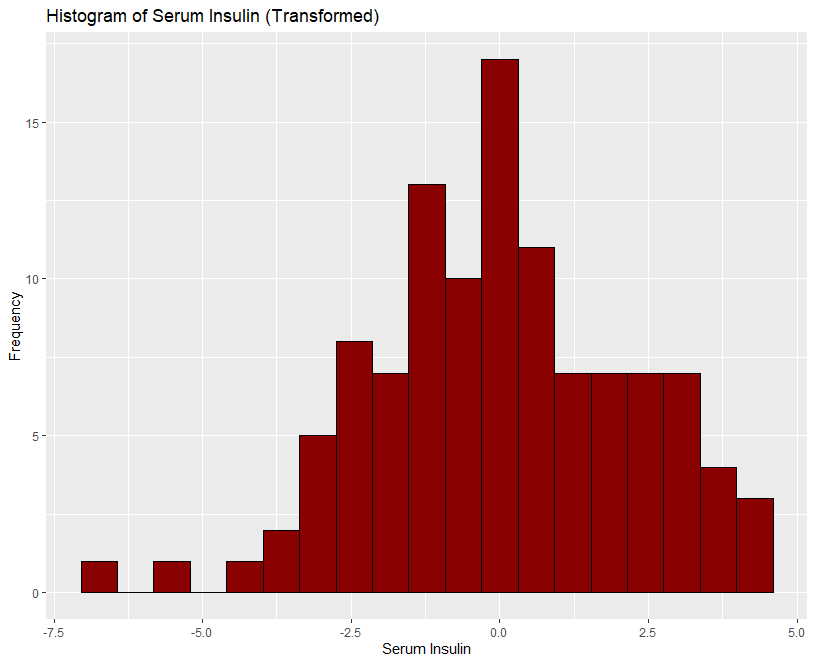


Supplementary Figure 5: Distribution of Transformed Data for Serum Insulin


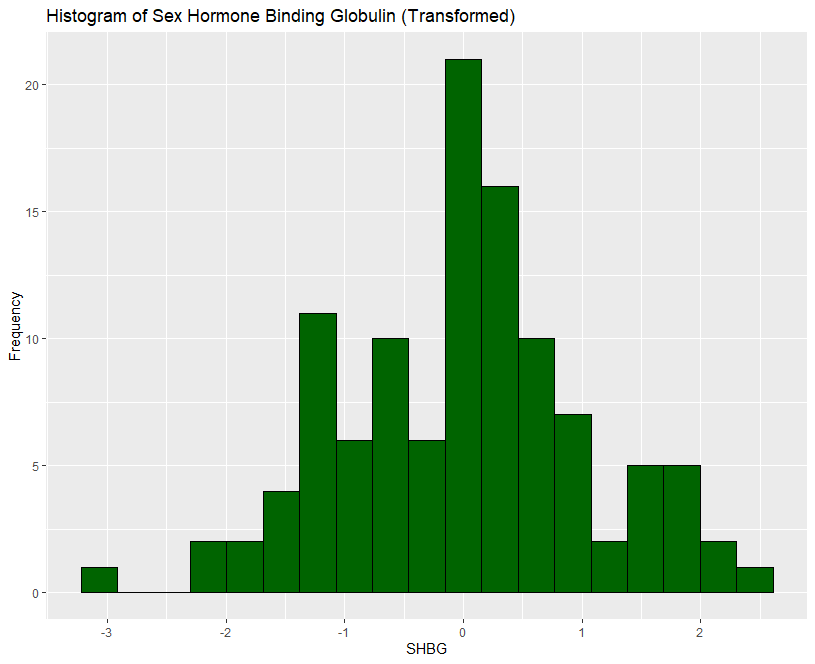


Supplementary Figure 6: Distribution of Transformed Data for Sex Hormone Binding Globulin
